# Multiple intracranial cavernomas with focal amyloid deposition – diagnostic pitfalls 

**DOI:** 10.5414/NP300397

**Published:** 2011-10-18

**Authors:** T. Velnar, G. Bunc, D. Flisar, D. Kulaš, A. Woehrer, H. Budka, M. Popović

**Affiliations:** 1 University Medical Center Maribor, Maribor, Slovenia,; 2 Institute of Neurology, Medical University of Vienna, Vienna, Austria,; 3 Institute of Pathology, Faculty of Medicine, Ljubljana,; 4 University Medical Center, Ljubljana, Slovenia

**Keywords:** intracranial cavernomas, amyloid, biopsy, neuroimaging

## Abstract

We report a case of a patient with multiple, intracranial superficial calcified tumorous lesions with focal amyloid deposition. On the basis of the first neuronavigated needle biopsy, the tumors were originally assessed as amyloidomas. Additional data was obtained from a second biopsy and supplementary neuroimaging information and the tumors were diagnosed as of vascular origin, probably cavernomas. The report exemplifies how only one diagnostic tool may sometimes be misleading in establishing a final diagnosis. The additional imaging may thoroughly enhance, supplement and improve the diagnostic process.

## Introduction 

The brain can be affected by a varity of disorders associated with amyloid deposition [1]. Rarely, intracranial mass lesions containing amyloid may be found, occurring either in association with focal amyloid angiopathy and called amyloidomas or as smaller amyloid deposits in the vessel walls of vascular malformations [1, 2, 3]. We report a case of a female patient with multiple, intracranial superficial calcified tumorous lesions with focal amyloid deposition. On the basis of the first neuronavigated needle biopsy, the tumors were originally assessed as amyloidomas. After additional data was obtained from a second biopsy and supplementary neuroimaging information, the tumors were diagnosed as of vascular origin, probably cavernomas. 

## Case report 

**Figure 1.**
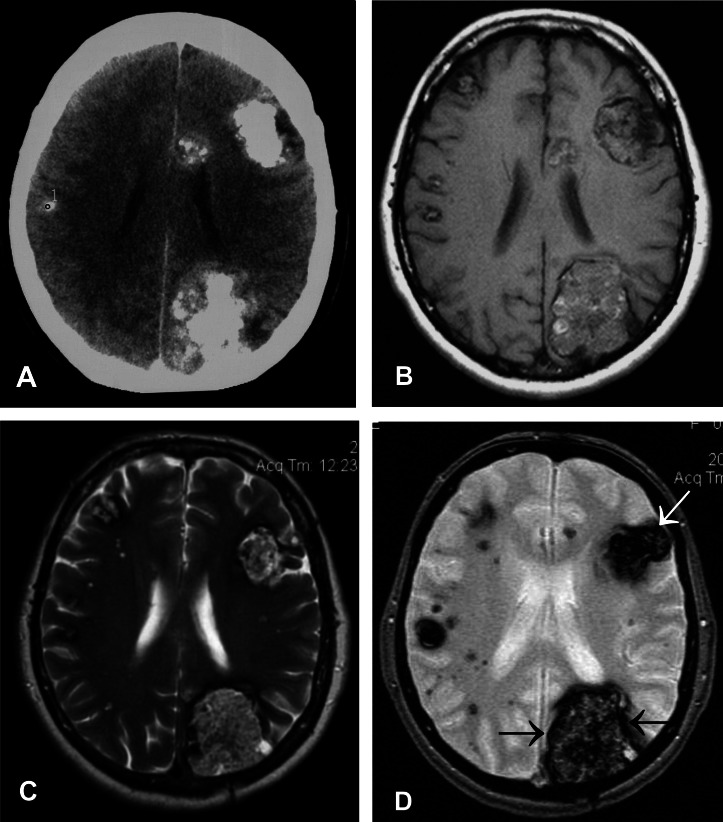
Neuroradiological feature of multiple cavernomas. The axial CT scan shows hyperdense expansive multifocal leptomeningial lesions. The signal intensity arises from intralesional calcium deposits (A). T1 weighted MRI shows two more superficial lesions. The intensity of signal is uneven and only focally hyperintensive (B). The T2 weighted MRI demonstrates the same lesions as in T1 and some smaller lesions scattered inside deep brain tissue, all of them showing hyperintensive but uneven signal (C). The T2* MRI shows even more small lesions in the deep brain tissue, giving a hypointensive signal. The larger superficial tumours are unevenly hypointensive and encircled with a thin hypointensive rim that corresponds to hemosiderin deposits located in and around the lesions (arrows) (D).

A 46-year-old female patient with unremarkable medical history was admitted to the neurology department in 2005 due to a first grand-mal epileptic seizure, which was successfully treated by intravenous application of diazepam. No abnormalities were found during the neurological examination, with the exception of a partial lower right quadrantanopia on confrontational visual field testing. A native CT scan of the brain revealed multiple calcified tumors in both cerebral hemispheres, without surrounding edema (Figure 1A). On a contrast CT scan, no additional enhancement of the lesions was seen (figure not shown). In addition to the superficial larger tumors, MRI disclosed smaller foci scattered in the deep brain tissue, all hyperintensive on T2 weighted sequences and hypointense on T1 weighted sequences (Figure 1B, C). 

**Figure 2.**
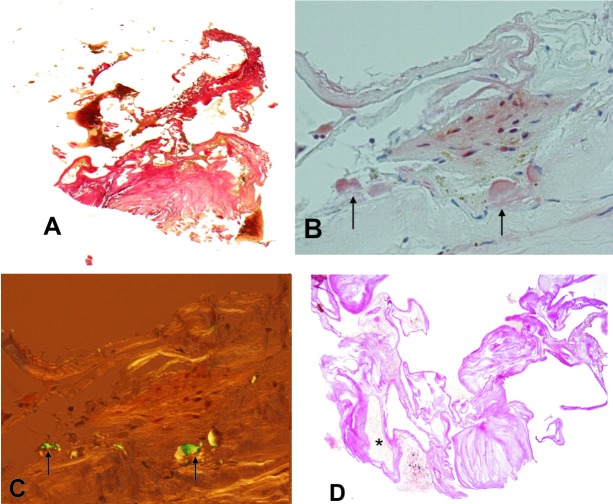
Two biopsies of the largest occipital tumor. A: The first needle biopsy, van Gisson: Amorphous, almost acellular, eosinophilic tissue without obvious vessel formation. B: Two foci of congophilic material (arrows) with a characteristic green birefringence under the polarised microscope (arrows) in C. The second biopsy of the same tumor, van Gisson: Several blood-filled vascular channels (asterisk) separated by fibrotic hyalinised tissue (D).

A small needle biopsy of the largest occipital tumor revealed hypocellular connective tissue with focal tinctorial and optical characteristics of amyloid deposits (Figure 2A, B, C). Immunohistochemistry with antibodies against amyloid b, immunoglobulin light chains, amyloid associated protein and transthyretin (TRT) showed diffuse labeling of the tissue with antibodies against both light chains and TRT, which was considered non-specific. A few months later, the patient underwent an open biopsy of the same stony-hard tumor in order to get an additional, rather small amount of tissue. Microscopic examination of the second biopsy revealed dilated blood spaces, separated by unevenly thick, hyalinized and focally calcified connective tissue, consistent with a diagnosis of a vascular malformation, most likely cavernous hemangioma (Figure 2d). No amyloid was present in the specimen. This finding prompted further radiological examinations on T2-weighted gradient-echo (T2*) MRI (Figure 1D), which supported the microscopic diagnosis of a vascular malformation. In addition, the hypointense peritumoural rim, irregular hypointense intratumoural foci corresponding to hemosiderin deposition, and the multilocular distribution of small hypointense foci supported the diagnosis. The patient is currently in good condition without neurological deficits or symptoms. The control MRI did not show any change. 

## Discussion 

The most common form of amyloid deposits in the brain is cerebral amyloid angiopathy (CAA), with or without Alzheimer’s neurodegenerative changes, preferentially affecting the cortical and leptomeningial blood vessels. CAAs do not usually show any changes on neuroimaging but rare pseudotumors in association with CAAs have been reported [4, 5]. In cerebral amyloidomas, in contrast, amyloid angiopathy is focal, usually of periventricular location, presenting as mass lesions [1, 6, 7]. In addition to amyloid deposits, focal calcifications may also be present in amyloidomas [1, 6]. Since only a small part of the tumor tissue can be achieved by needle biopsy, as in our case, we believe that various proportions of amyloid deposited in the vessel walls, which were randomly scattered across different regions of the tumor, may therefore be encountered in amyloidomas. 

The second small biopsy of the stony-hard tumor in the right occipital lobe revealed the vascular nature of the lesions. Calcified cavernomas are the most likely possibility, on the basis of the negative three-dimensional CT angiography and T2* MRI [8, 9]. Since cavernomas have no arteriovenous shunts, they are angiographically occult [10]. In contrast to CT, which nicely detects fresh bleeding and calcifications, the MRI technique has been recognized as a useful tool for diagnosing even small cavernomas and old intracranial hemorrhages with hemosiderin build-up [8, 9, 10]. In doubtful cases, T2* MRI is especially helpful. Amyloid that may be randomly deposited in pre-existing vascular malformations, as happened in the first biopsy in our case, can be misleading, especially if the biopsy is tiny. This may therefore mask the histological diagnosis of the lesion, especially in the absence of characteristic features of a vascular malformation. Even though CT and MRI scans of amyloidomas have typical features, the diagnostics should be supported by microscopic examination [6]. On a CT scan, amyloidomas appear as solitary or multiple hyperintense nodules with irregular margins, located preferentially in the subventricular white matter, with little or no mass effect on the surrounding brain parenchyma. The tumors are enhanced after application of gadolinium contrast [1, 6]. Cavernomas appear as hyperintense or heterogeneous lesions with calcifications and a variable degree of peripheral contrast enhancement on a CT scan [10]. MRI studies show well-defined lesions, sometimes with hemorrhage, which may be surrounded by peripheral edema. Hemosiderin deposits resulting from old hemorrhage may produce a low peripheral and heterogeneous central signal on both T1- and T2-weighted images [11]. 

Despite the presence of multiple intracranial masses, the patient was asymptomatic, except for an epileptic seizure and visual field defects. This suggests that the tumors were longstanding, with little or no mass effect on surrounding structures. Supratentorial cavernomas may be associated with chronic epilepsy, which is generally the most frequent manifestation of the disease. 

The most common complication of cavernomas is bleeding, resulting from rupture of ectatic blood vessels [9, 11]. In comparison with AVM, bleeding in cavernomas is not as instant and as devastating but might recur over an extended period of time. Extralesional blood is resorbed with time, leaving a rim of hemosiderin pigment. Intralesional bleeding might lead to the formation of larger collections that often calcify [11, 12]. Frischer et al. [3] reported that the histology of cavernomas is not diagnostic in 18.4% of cases, probably due to symptomatic hemorrhage, which destroys the vascular malformation. The tissue attained during the first needle biopsy was not diagnostic for a cavernoma and the focal amyloid deposits represented a diagnostic pitfall, which lead to an incorrect diagnosis. In such cases, the radiological signs of peritumoural hemorrhage are particularly useful, since histological examination may not lead to a straightforward and definite diagnosis of the lesion. Amyloid aggregates may be found in 16.1% of cavernomas deposited in and around vessel walls of highly variable thickness [3]. 

## Conclusion 

The present report illustrates how only one diagnostic tool may sometimes be misleading in establishing a final diagnosis and how imaging may thoroughly enhance, supplement and improve the diagnostic process. In our case, only a small needle biopsy of the focal amyloid deposits in multiple intracranial vascular tumors, which were probably cavernomas, was misleading. Additional biopsy and T2-weighted gradient-echo MRI were needed for accurate final diagnosis.
